# Optimizing spinal cord injury care in Canada: Development of a framework for strategy and action

**DOI:** 10.3389/fpubh.2022.921926

**Published:** 2022-11-07

**Authors:** Joanna Marie B. Rivera, Charlene Yousefi, Christiana L. Cheng, Cameron D. Norman, Jeanne Legare, Alana McFarlane, Vanessa K. Noonan

**Affiliations:** ^1^Praxis Spinal Cord Institute, Vancouver, BC, Canada; ^2^Cense Ltd., Toronto, ON, Canada

**Keywords:** spinal cord injury, health strategy, strategy development, health services for persons with disabilities, health care delivery, health communication

## Abstract

National health strategies are integral in defining the vision and strategic direction for ensuring the health of a population or for a specific health area. To facilitate a national coordinated approach in spinal cord injury (SCI) research and care in Canada, Praxis Spinal Cord Institute, with support from national experts and funding from the Government of Canada, developed a national strategy to advance SCI care, health, and wellness based on previous SCI strategic documents. This paper describes the development process of the *SCI Care for Canada: A Framework for Strategy and Action*. Specifically, it covers the process of building on historical and existing work of SCI in Canada through a thorough review of literature to inform community consultations and co-creation design. Furthermore, this paper describes planning for communication, dissemination, and evaluation. The *SCI Care Strategic Framework* promotes an updated common understanding of the goals and vision of the SCI community, as well as strengths and priorities within the SCI system regarding care, health, and wellness. Additionally, it supports the coordination and scaling up of SCI advancements to make a sustainable impact nationwide focusing on the needs of people living with SCI.

## Introduction

National health strategies are integral in defining the vision of a population and providing coordinated strategic directions to help achieve that vision (1). Health strategies have been developed and used to identify gaps in the health system, to bridge these gaps, and to ensure that innovations in care advance in a coordinated and evidence-based way (2). For spinal cord injury (SCI) in Canada, a national strategic plan offers the opportunity to provide a future-oriented road map to advance SCI care through a coordinated and collaborative approach.

During a meeting with a broad range of SCI stakeholders in May 2018, a proposal to develop a strategy to advance SCI care, health, and wellness was recommended to reflect ongoing work and facilitate a national coordinated approach for future planning. A national strategy can be a powerful agent of change to drive improvements in SCI care and align fragmented and traditionally siloed areas. The proposal was advanced by the Praxis Spinal Cord Institute (Praxis) with SCI experts providing recommendations on key elements to include in the national strategy. With further consultations and support from the SCI community and funding from Pacific Economic Development Canada (PacifiCan), formerly Western Economic Diversification through the Government of Canada, Praxis proceeded to convene stakeholder engagement sessions to co-create a national SCI strategy for collective impact (3).

Using a community case study approach, this paper describes the development of the national SCI Strategy report *SCI Care for Canada: A Framework for Strategy and Action* (*SCI Care Strategic Framework*) (4), and plans for dissemination and evaluation. Developing health strategies is an ongoing process that must be reflective and responsive to the needs of the local community and context. However, literature describing the process of developing health strategies remain scarce or publicly inaccessible. This paper provides an overview of the strategy development process and shares lessons learned.

For this paper, the following terminology is used:

- **Health/Wellness**: The World Health Organization describes health as “a state of complete physical, mental and social wellbeing, and not merely the absence of disease or infirmity” (5).
- **Health care/Care**: Services offered to preserve emotional and physical health and wellbeing; examples of health care settings include acute care facilities, long-term facilities, outpatient facilities, etc. (6).
- **Action plan:** Specific action points provided to achieve goals laid out in policies or strategies. Action plans often have a shorter time frame than strategies (e.g., 1–2 years) (7).
- **Framework:** Provides a common understanding of a health system, its gaps and challenges; and communicates and promotes areas and strategies for strengthening, enhancement, and monitoring to achieve specific outcomes (8).
- **Model of care/care pathway:** Pathways by which health care services are systematically delivered (9).
- **Strategy:** A plan of action or policy designed to achieve a major or overall goal (10). Strategies are long-term action plans for the future, usually covering three or more years, and focused on a particular goal (7).

## Context

In Canada, more than 86,000 people are affected by spinal cord injuries, from either a traumatic (e.g., fall) or non-traumatic cause (e.g., tumor) (11). SCI is a life-altering condition that significantly impacts one's daily life, physical and emotional health, and quality of life (9). Following an SCI, individuals require comprehensive care and support to maximize recovery and resume meaningful participation in everyday occupations and activities of daily living (12).

There have been great advancements in SCI research and care due to the effort of the SCI community (composed of researchers, clinicians, individuals living with SCI and family members, community organizations, administrators etc.), often working and collaborating multi-nationally (13). These advancements are made possible through leadership of individuals such as Rick Hansen with his Man In Motion World Tour in 1985–1987, when he wheeled over 40,000 kilometers around the world and raised more than $26 million to support SCI research and quality of life initiatives. This work continues to advance with an engaged SCI community working at the regional, provincial and national level and the involvement of organizations such as the Rick Hansen Foundation (today also focuses on accessibility), SCI Canada and SCI Provincial Organizations, Ontario Neurotrauma Foundation (ended in March 2021) and Praxis Spinal Cord Institute (formerly known as the Rick Hansen Institute).

In 2003, Rick Hansen Foundation supported a Cross Canada Checkup, a national report which identified gaps and potential solutions to accelerate improvements in the quality of life of people with SCI (14). The desired outcome was “to develop a Network driven by consumers with SCI and committed to a shared vision and breakthrough solutions that will accelerate improvements in quality of life of people with spinal cord injuries” (14). The importance of “a shared national vision and direction for the SCI community” and “unifying and leveraging resources across the SCI community to support key priorities” highlighted in this foundational report led to a number of pivotal white papers on priority areas. As an example, a national environmental scan on current practice and capacity in SCI rehabilitation (15) informed the development (16, 17) and subsequent implementation of indicators to improve SCI rehabilitation care (18). Furthermore, partnerships among Canadian SCI stakeholders have resulted in initiatives such as the production of Integrated Knowledge Translation Guiding Principles for conducting and disseminating SCI research in collaboration with research users (19), the development of the national Rick Hansen SCI Registry (RHSCIR) (20), and the implementation of Acute and Rehabilitation SCI Standards in RHSCIR acute and rehabilitation SCI centers across Canada (21).

However, health system gaps for SCI care and inconsistencies in ensuring holistic health and quality of life for people living with SCI remain (14). The provision of SCI care and support spans the life course of an individual, although, both specialized and primary care are not yet standardized and equitable access to quality health care and support remains a challenge for many Canadians living with SCI (9). Additionally, regional disparities related to availability and access to SCI persist (14, 22). For example, rural areas are more likely to lack specialized care for persons with SCI resulting in the reliance on non-specialized or primary care physicians who may lack SCI knowledge or expertise (23). With many SCI services concentrated in urban centers (22), this can impact an individual's decision to relocate or remain in city centers to be able to access needed health services.

Further, gaps remain in facilitating the transition between discharge from the hospital and return to the community (22). Individuals and their families feel like they are returning to a new reality of living with a SCI. While many community organizations exist to provide necessary support and services for daily and community living and to help develop community connections (e.g., providing peer support, employment services, and other resources), many individuals may still experience difficulties in obtaining essential support for home and community living. Such supports could include accessible housing, equipment and technical aids, and transportation (24). Eventual return to education or employment can also be an added challenge due to a myriad of factors (25).

Not only must the health system be responsive to present needs, but it must anticipate and be resilient to future trends and events, both at the macro level (e.g., future pandemics, climate events) (26) and meso and micro levels (e.g., increasing need for more integrated care, smoother care transitions from primary care to community, and management of long-term care needs) (27). Mutual understanding, concerted effort, and multi-stakeholder collaboration are imperative in providing the necessary services and supports to address the current and future needs of individuals living with SCI so that they can live their best life following an injury. In Canada, a learning health system model, whereby data and experiences are integrated with evidence to produce quality care (28) has been proposed for SCI (29) and adopted provincially (30) to achieve this vision.

Based on the RHSCIR data, it is evident that the SCI population is changing. On average, individuals acquiring a traumatic SCI have become older. In 2004 the mean age at injury was 45 years old compared to 2019 where the mean age at injury was 52 years old (31). Furthermore, persons are now more likely to acquire an injury from falls compared to transportation. There is also a shift towards individuals sustaining more incomplete injuries versus complete injuries. It is forecasted that in 2032, the median age of injury will increase to 57 years old and persons over the age of 60 will account for 46% of all new injuries. Care costs will increase by 54% and rest-of-life costs will increase by 37%, requiring an additional $16.4 million (32). Therefore, it is critical that changes in demographics and management of SCI be considered when planning for current and future health care delivery needs.

## The *SCI Care Strategic Framework* and the development process

This section provides an overview of the strategy development process from its inception to future evaluation plans. Key elements of the process are described below including background research and groundwork, consultation design, and launch of the *SCI Care Strategic Framework*. Core components of the Framework are then summarized followed by a description of communication and dissemination activities, and evaluation plans.

### Co-creation, community consultations, and iterative design

The journey toward *the SCI Care Strategic Framework* began in 2018 with initial discussions around developing a national SCI care strategy that would ensure the integration of initiatives related to key elements such as care, research and innovation, policy and advocacy and propose a national leadership advisory group for implementation. In 2018 Praxis, with support from the external consultation team Cense Ltd., convened a series of key activities including stakeholder consultations and engagement, strategic planning, and research. This multi-year process included the release of the 2019 report *Being Bold: Toward a National Spinal Cord Injury Care, Health & Wellness Strategy* (*Being Bold*) (10) and culminated in the release of the *SCI Care in Canada: A Framework for Strategy and Action* report (*SCI Care Strategic Framework*) in 2021 (4).

Various planning activities and extensive background research (including academic and gray literature reviews) supported the strategy development process. An in-depth stakeholder map was completed, which included national and multi-sectoral actors within the SCI community and the broader ecosystem of clinical sites, research networks, government ministries, third-party insurance, advocacy groups and other community-based organizations related to disability and SCI. This stakeholder map was stratified by province or territory and used to support invitations to consultation activities. Further, stakeholder engagement and communication plans were developed to identify, prioritize, and plan for engagement activities. Concurrently, various literature searches, reviews of reports, research articles and white papers were conducted to better understand the landscape of SCI care in Canada and to supplement the findings from earlier national consultations (14, 17, 33–36). A search of academic and country databases provided a basis of understanding for published or publicly available international SCI strategies and national plans. Table 1 provides examples of relevant international and national SCI strategies and planning documents. Finally, a review of Canadian health strategies helped to elucidate key aspects and levers of national health strategies. Most notably, co-learnings from cancer (43), mental health (44), stroke (45), and dementia (46) highlighted the importance of iterative public engagement and collaborative networks for stakeholder consultations, community knowledge sharing and cross-sectoral support.

**Table 1:** Examples of SCI strategies and national plans.

**Country/ Region**	**Document type**	**Document (type and title)**	**Description**
United Kingdom	Clinical protocols	Management of People with Spinal Cord Injury (2011) (37)	Various elements of a National Care Pathway, including SCI joint protocols and clinical policies were developed by The Spinal Cord Injury Strategy Board (38).
Queensland, Australia	Model of care framework	Queensland Spinal Cord Injuries Service Model of Care framework (2018) (39)	This model of care “provides a plan for health care for individuals with spinal cord injury. It describes the aims and principles of service delivery, the evidence-based practices and frameworks used at QSCIS and the flow of patient services as they progress through the continuum of care” (pg. 5).
	Health service plan	Statewide Adult Spinal Cord Injury Health Service Plan 2016-2026 (2016) (40)	Builds on the Model of Care Framework “by identifying opportunities for improving services for people with SCI, their families and the community” (pg. v).
New Zealand	Action plan	Spinal Cord Impairment Action Plan 2014-2019 (2014) (41)	The Action Plan “should be used as the basis for more detailed plans that will be developed and implemented by the lead agencies identified” (pg. 4).
Canada	Network and strategy planning	Rick Hansen SCI Network. Cross Canada Checkup. Interim report of the national consultations on SCI services in Canada - a qualitative overview (2004) (14)	A national review and consultation to “examine the current state of treatment, support and community services for people with SCI. The Cross Canada Checkup is an important foundation report to help identify and develop breakthrough solutions that address the priority needs of people with SCI and bring the SCI community together working toward a shared national vision” (pg. 92).
			Note: the Cross Canada Checkup reviewed previous SCI planning documents and the findings informed multiple white papers and subsequent initiatives.
Alberta, Canada	Strategy	The Spinal Cord Injury Strategy for Alberta (2021) (42)	A Provincial Strategy that “will facilitate collaboration and connection between these core sectors to provide concrete and measurable recommendations, identify medical and social best practices, and support innovative medical, technological and social interventions” (pg. 2).

Consultations occurred in phases throughout the strategy development process and involved a broad range of stakeholders including individuals living with SCI and their families, clinicians, researchers, community partners, administrators, organizations with experience developing strategies, innovators etc. This provided an opportunity to hear diverse perspectives and engage groups throughout the country. Invitations to participate in the consultation process were widely distributed through SCI-related networks and social media channels. Several platforms were used to engage with the participants including a Canada-wide webinar consultation, an online survey, and solicitations for feedback through the circulation of a 2-page draft document with a feedback form. Data from a national needs assessment developed in partnership with Health Standards Organization was also used to develop the Framework document.

The 2019 *Being Bold* report was published after the first phase of initial exploratory consultations and summarized consultation and research findings around the interest and feasibility of developing a national SCI strategy (10). The report identified support from the community and interest in co-creating a path forward with clear recommendations that could be further refined, developed, and implemented by the SCI community within their local and regional context (10). The community recommended that future plans must build on existing accomplishments and efforts of the SCI community, given the historical work and planning that has been done throughout the years, and that there was a determined desire to act and make change. It was recommended that the strengths and areas of need in the community be identified to advance a future vision of SCI care, health and wellness across the country. Additional recommendations included co-creating from the start, bringing in diverse voices and perspectives, engaging with a variety of actors while leveraging existing activities and resources, and continuously communicating progress (10). Thus, informed by the consultation report, Praxis and Cense Ltd., proceeded with the next steps to develop an actionable framework to form the basis for a national SCI strategy which included goals, tactics, and strategies to achieve a collective vision set for the next 10 years (10). Praxis served as a backbone organization (convenor and facilitator) for the SCI community in its development.

### Launch of the *SCI Care Strategic Framework* and key components

Building on community consultations, a review of literature, and existing national consultation documents (e.g., the Cross Canada Checkup), the *SCI Care Strategic Framework* was published in 2021. The Framework recommends a coordinated approach to advancing care in Canada (4) to achieve the strategy's shared vision for “a timely, human-centered, accessible, equitable, and high-quality system of care driven by evidence and nationally and internationally recognized for its excellence, innovation, and outcomes across the life course” (4).

**Figure 1:**
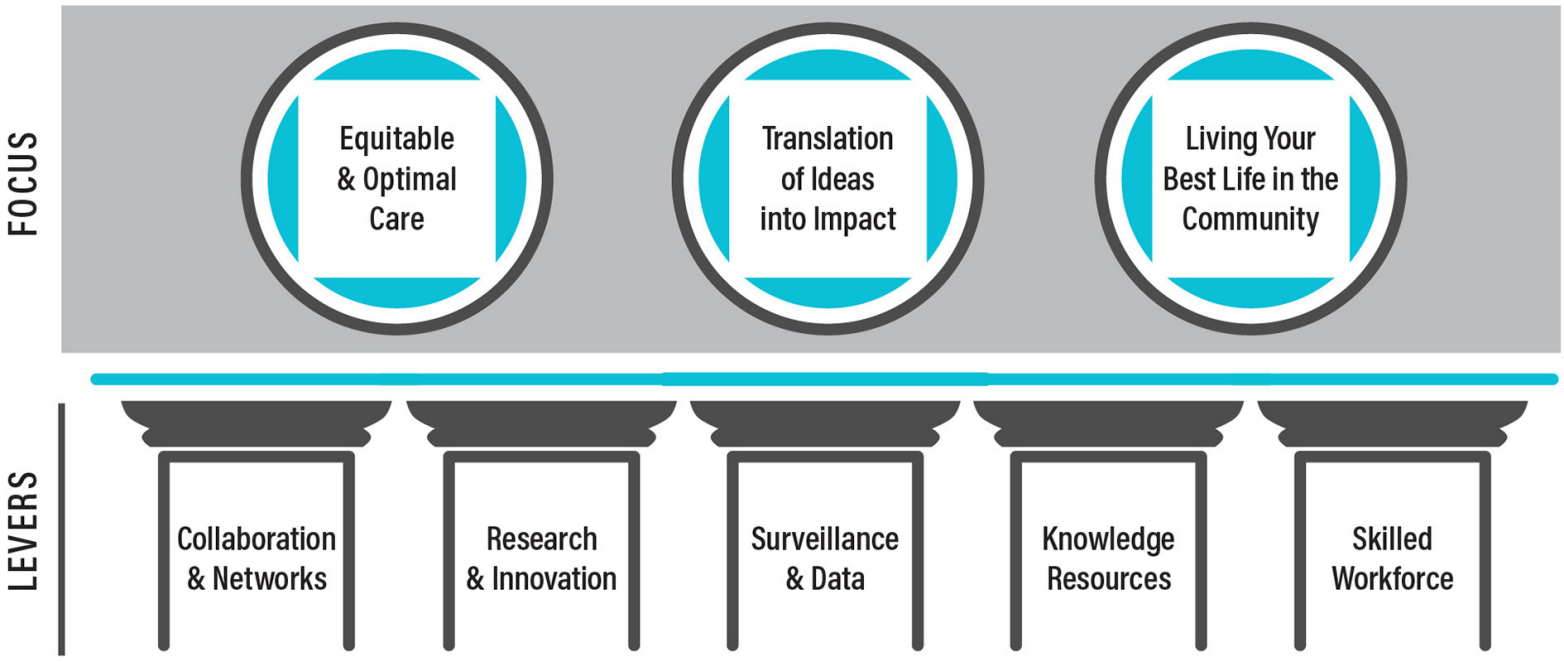
The three areas of focus and five pillars in the *SCI Care in Canada: A Framework for Strategy and Action*.

The *SCI Care Strategic Framework* outlines three key areas: 1) equitable and optimal care, 2) translation of ideas into impact, and 3) living your best life in the community; and five levers or pillars of activity: 1) collaboration and networks, 2) research and innovation, 3) surveillance and data, 4) knowledge resources, and 5) skilled workforce, which serve as the system levers for transforming SCI care (Figure 1).

- ***Equitable and optimal care*** ensures best possible health outcomes for people with SCI including the need to better align fragmented systems of SCI care. Examples of initiatives to achieve this include continue supporting the development of national quality indicators (16) and outlining an SCI Model of Care for Canada, proposed by Ho et al. (9).
- ***Translation of ideas into impact*** emphasizes the importance of translating research evidence into implementation and real-world outcomes. It takes an estimated average of 17 years for research evidence to be adopted by health professionals and the public (47), a translational gap that has implications for health care users, current health programming, and implementation of best practices including innovations and technologies. Examples of initiatives to achieve this include commercialization programs to facilitate the development of prototypes and reimbursement pathways for companies to ensure individuals with SCI benefit from the innovation (48).
- ***Living your best life in the community*** highlights the need to have integrated community-based systems and supports in place to ensure that people living with SCI can access appropriate and quality community, social, and health care and services when and where they need it. Examples of initiatives to achieve this include developing a peer-led health coaching program to support health and wellness in the community (49) and peer mentorship programs implemented by community-based SCI organizations (50).

### Dissemination, communication, and impact of networks

In 2020, planning for dissemination and communication of the *SCI Care Strategic Framework* began and occurred concurrently with the writing of the document. Feedback from the consultations included the need to further support implementation of best practices and to make communication a priority. Communication is an ongoing process of continuously planning and building momentum while adjusting activities to respond to real-time feedback. As such, a robust communication plan was developed including creation of digital content that could be shared through various health communication channels and networks such as Praxis website, newsletters, and social media.

Learning Circles (knowledge exchange events that focus on storytelling and encourage collaborative learning) were developed to enhance knowledge sharing on current and emergent SCI topics of interest while promoting the launch of the Framework. Learning Circles are activities guided by values of collaboration, openness and sharing, partnerships, supported learning, and commitment to excellence for the vision of SCI care in Canada and can take the form of presentations (e.g., webinars, in-person meetings) or soundbites. They provide opportunities to share knowledge, support connection and communication, showcase innovative projects from across the country, and start conversations on topics salient to the SCI community. For example, an on-line webinar called the “Fireside Chat on Indigenous Disability Awareness Month” was open to the SCI community. This event included a facilitated storytelling session guided by leaders from the Indigenous SCI community followed by an open discussion to understand the challenges faced by Indigenous peoples with SCI and future directions. Learning Circles are also part of newsletters (including video clips and resources) where Praxis shares best practices that are being championed by individuals and organizations across Canada to showcase excellence in SCI research, care and innovation in action, with the goal of promoting uptake and collaboration.

Moreover, dissemination and communication plans also included engaging and fostering collaborative networks. Networks with common goals as the Framework can support realization and implementation of its recommendations, tactics and strategies across regions. Knowledge sharing can also be enhanced by galvanizing these networks, which are often led by champions who have a passion for and see tremendous value in engaging community members with a common purpose.

There is a history of SCI networks leading collaborative work to effect positive change and advance SCI in Canada. An example is the SCI Knowledge Mobilization Network (SCI KMN), a network that adapted and implemented best practices to prevent and manage pressure injuries and pain after SCI (51). SCI KMN morphed into a larger quality improvement collaborative, the Spinal Cord Injury Implementation, Evaluation and Quality Care Consortium (SCI IEQCC), which supports the implementation of best practice indicators and interventions in domains such as emotional wellbeing, sexual health, wheeled mobility, walking, and urinary tract infection and produces report cards to evaluate the impact. Originally started in 5 rehabilitation centers in Ontario (18) with support from the Ontario Ministry of Health, the SCI IEQCC is now also supported by Praxis to improve the implementation of best practices in Edmonton, Calgary, Halifax, Fredericton and Charlottetown. Additionally, recognizing the importance of networks, Praxis launched the network development grant competition to provide seed funding to existing and emerging Canadian networks to nurture the development and/or sustainability of network activities (52).

### Evaluation

Evaluation is an essential component of important health interventions and programming (53), as such it was included as a key activity during the initial planning. The *SCI Care Strategic Framework* utilizes a Development Evaluation, an approach designed to create a system of learning and action within the project (54). This approach provides a means to gather real-time data, ensures ongoing documentation of activities, and creates a feedback mechanism to inform the evolution of the *SCI Care Strategic Framework*. Supplementary Table 1 outlines the logic model which describes examples of the Framework's activities and intended outcomes for evaluation purposes.

Two evaluations will be conducted during mid-term and end-term time points. The first is a process evaluation to assess the development and dissemination of the *SCI Care Strategic Framework* across Canada through various knowledge sharing activities. The second is an impact evaluation designed to assess effectiveness in achieving the goals and vision of the Framework. The evaluation will be governed by an independent Evaluation Advisory Committee, supported by contracted external evaluators, and will use a person-centric/human-centric approach focusing on positive changes for people with SCI.

## Discussion

Flexibility and adaptability were important throughout the strategy development process. The multi-year process didn't happen in a vacuum and was impacted by events both seen and unforeseen. Thus, having the latitude and ability to adapt plans greatly impacted the success of the Framework launch. Flexibility was necessary to ensure that community feedback was continuously integrated into the Framework. An important development driven by consultation feedback was the shift from creating a national strategy to instead developing a framework. This shift was due to feedback on the potential difficulties of having a broad national strategy which could miss the unique opportunities, challenges, and needs of regions and local communities. Therefore, the result was to develop a framework for strategy and action. As a Framework, it is flexible in adapting to meet the emerging needs and shifts in systems of care, services and support. It provides clear guidance without being too prescriptive to allow partners, organizations and stakeholders the autonomy to make modifications based on regional needs and opportunities. Additionally, the Framework forms the basis for a national strategy while building on what is being done, facilitating sharing the success of best practices to support spread and coordination of efforts while guiding and aligning various proposals at provincial and national levels.

Additionally, adaptability and flexibility in the face of unforeseen world events were put to the test as COVID-19 was declared a pandemic (55) in the midst of developing the *SCI Care for Canada: A Framework for Strategy and Action*. Regional and national resources and focus shifted toward addressing the challenges posed by the pandemic and new digital ways of communication and consultation needed to be designed. The strategy development team had to revise initial communication and engagement plans and explore more online activities and their feasibility with target audiences, such as the creation of Learning Circles as a key knowledge-sharing tool.

Community engagement and communication are core to the strategy development process, but consultations take time and can be long and very involved. Moreover, the SCI community in Canada is relatively small and care needed to be taken to balance gathering as much of the necessary feedback and data needed with consultation fatigue. Thus, new ways of engaging had to be developed to include a broad range of SCI stakeholders such as sending email invites to attend online Framework consultations to stakeholders from the health ministry level to local SCI community organizations across Canada.

Aligning and supporting initiatives and projects with common goals to the Framework would support its implementation and can foster engagement with research and clinical stakeholders. For example, concurrent with the development of the strategy process, networks of various SCI stakeholders and relevant organizations formed regionally and nationally to a) provide coordinated and person-centered services to support the care, health, and wellbeing of individuals as they transition across the continuum of care and return to their communities; b) provide education, knowledge exchange and capacity building opportunities for those involved in the provision of services and supports for people with SCI; and c) facilitate and champion practice and policy changes to optimize the local delivery of services for people with SCI. SCI communities are collaborating outside their own discipline, organization and/or region to find collaborative solutions to complex problems, to learn from each other and build specialized skills to better support people with SCI, and to better coordinate care, services and support across settings.

In addition to leveraging current networks, alignment with new provincial initiatives is key to the success of the Framework. The timing is good as work is underway in Alberta on their provincial SCI Strategy (42) and a transition in care model using hubs and spokes system (56), and in Ontario on defining the ideal neurotrauma care pathways. Lessons learned from these initiatives will be informative for the Framework to scale up across the country to improve SCI care.

Furthermore, to support communication and knowledge sharing, there is a need to find ways to share Canadian SCI resources across the continuum of care and across the county. While valuable SCI resources exist across the country, sharing of resources is often limited by region or within disciplines of practice or areas of interest. For example, the SCI Community Interactive Webinars Series put on by SCI Alberta (57) and the webinar series by the Circulus SCI Primary Care Network (58) both present excellent evidence-based resources for the SCI community in an accessible YouTube video format but might not be well-known outside of their region or discipline, respectively.

Building internal momentum within the strategy development team was possible by having dedicated team members who were able to focus on supporting the strategy as a key project. Additionally, the majority of the strategy development team, including the external consultants, were available for most of the development processes. Having external consultants outside of the SCI community allowed them to be objective and remain at arm's length while providing continuity and building rapport with SCI community members throughout the consultation process. Routine pre-scheduled meetings were also easy and effective ways to stay on track and check-in through the ebbs and flows of the development process.

### Conceptual or methodological constraints

The task of developing a national strategy without a specific fund or national body directing the process is atypical and presented methodological constraints. Among the most central of these was a need to engage organizations who were partners of Praxis in an activity that was to be shared in its benefits while adhering to the funding and resource commitments of Praxis. This meant ensuring that everything was done in a transparent manner and that parties across the SCI spectrum were aware of the purpose, intent, and strategy development process. Due in part to the inability to travel and canceling of in-person events due to COVID-19, some relevant parties may have been missed or were unable to be more strongly engaged during the development of the strategy. Early communication plans included attending conferences across Canada during pre-conference meetings as additional consultation events though, given COVID-19 measures at the time, these plans had to be reimagined as online activities. While online communication and social media fosters the opportunity to reach and engage people, there can be trade-offs with in-person consultations. Another contributing constraint was limited time and attentional resources available from experts and collaborating organizations to contribute to the process. Future plans include continuing to share and animate the *SCI Care Strategic Framework* to further engage the SCI community and advance its implementation.

## Conclusion

The *SCI Care for Canada: A Framework for Strategy and Action* promotes a common understanding of goals and vision of SCI in Canada as well as strengths and priorities within the SCI system of care, health, and wellness. Throughout the consultation and development of the Framework, a shared aim expressed by members of the SCI community was to ensure the best possible health and wellness for people living with SCI. Given the known gap in translating knowledge to clinical practice and real-world settings, the SCI community has stressed the urgent need for more timely translation and implementation of existing knowledge and emerging innovations. Coordination, collaborative effort, and dedicated resources are needed in continuing to provide accessible and equitable quality SCI care and scale up of SCI advancements to make a sustainable impact nation-wide focusing on the needs of people living with SCI.

## Data availability statement

The original contributions presented in the study are included in the article/Supplementary material, further inquiries can be directed to the corresponding author.

## Author contributions

JR drafted the manuscript. All authors contributed to the conception, design, development, dissemination of the *SCI Care Strategic Framework*, and critical revisions and final approval of the manuscript.

## Funding

This work is supported by the Praxis Spinal Cord Institute, Health Canada, Western Economic Diversification Canada, and the Governments of Alberta, British Columbia, Manitoba, and Ontario.

## Conflict of interest

Authors CN and JL were employed by Cense Ltd. and were contracted by Praxis to develop the *SCI Care Strategic Framework* but devoted their own time to this manuscript. The remaining authors declare that the research was conducted in the absence of any commercial or financial relationships that could be construed as a potential conflict of interest.

## Publisher's note

All claims expressed in this article are solely those of the authors and do not necessarily represent those of their affiliated organizations, or those of the publisher, the editors and the reviewers. Any product that may be evaluated in this article, or claim that may be made by its manufacturer, is not guaranteed or endorsed by the publisher.

## Supplementary material

Supplementary Table 1 Logic Model for Spinal Cord Injury Care Strategic Framework: Examples of activities and their links to outputs and outcomes.

## Abbreviations

IKT: integrated knowledge translation
RHSCIR: Rick Hansen Spinal Cord Injury Registry
SCI: spinal cord injury
SCI IEQCC, Spinal Cord Injury Implementation: Evaluation and Quality Care Consortium
SCI KMN: Spinal Cord Injury Knowledge Mobilization Network
WHO: World Health Organization.
