# Supplementary material for: Optimizing spinal cord injury care in Canada: Development of a framework for strategy and action
Source: Front Public Health. 2022 Nov 7;10:921926. doi: 10.3389/fpubh.2022.921926 (PMC9678047; doi:10.3389/fpubh.2022.921926)
Supplement: Supplementary Table 1 — Logic Model for Spinal Cord Injury Care Strategic Framework: Examples of activities and their links to outputs and outcomes. [file Table_1.pdf]

Supplementary Table 1: Logic Model for Spinal Cord Injury Care Strategic Framework: Examples of activities and their links to outputs and outcomes

Inputs	Near Term Activities (Apr 2021-Mar 2023)	Mid-Term Activities (Apr 2023- Mar 2028)	Outputs	Immediate Outcomes	Intermediate Outcomes	Long-Term Outcomes
SCI stakeholders<br><br>Funding	Establishing Pan-Canadian advisory networks to guide the strategy and promote healthcare partnerships to realize this vision and sustain the progress throughout the next decade	Establish partnerships with allied organizations on areas of shared interest, particularly home and community care to generate full-spectrum support for SCI across the life course	#/type of networks and partnerships established or accelerated<br>#/type of activities undertaken by above<br>Examples of engagement with people with lived experience	Enhanced Collaboration and Networks		Vision:<br>A timely, human-centred, accessible, equitable, and high-quality system of care driven by evidence and nationally and internationally recognized for its excellence, innovation, and outcomes across the life course.<br><br>**In 10 years, Canada will lead the world in SCI care.**
	Accelerating the formation of regional networks to connect the best practice and research groups together with people with lived experience				More Equitable and Optimal Care	
	Investing in the SCI national standards, best practices, and guidelines initiatives to advance excellence	Expand and enhance primary care SCI support to broaden access for people in every community in Canada	#/type of investments made, research completed, knowledge translation products developed and used	Enhanced Research & Innovation		
	Elevating the research on neuro-restorative and functional therapies to improve and save lives					
	Promoting incubator, innovator and accelerator programs aimed at technology transfer and commercialization to get the best ideas into the market	Develop commercialization pathways connecting R&D and clinical care research to create value that converts to clinical excellence and innovative products	#/type of innovations/ technologies incubated/ accelerated<br># SMEs assisted		Enhanced Translation of Ideas into Impact	
	Advancing the national data strategy by optimizing the core infrastructure and processes which will expedite the translation of research to practice	Further honing a maturing and responsive data strategy as needs and opportunities evolve	Annual updates on national data strategy	Enhanced Surveillance & Data		
	Raising the profile of the educational and peer-outreach programming developed for and by those with lived experience		Uptake of education and peer-outreach programming	Enhanced Knowledge Resources	Improved Living of Best Lives in the Community	
	Conducting an environmental scan of existing policies for access to equipment and services in the community across provinces/territories	Creating evidence-based resources and working with partners to promote equitable access to supplies and services in the community across provinces/territories	Annual updates on changes to policies or increases in access to services or equipment			

Inputs	Near Term Activities (Apr 2021-Mar 2023)	Mid-Term Activities (Apr 2023- Mar 2028)	Outputs	Immediate Outcomes	Intermediate Outcomes	Long-Term Outcomes
		Recruit next-generation leaders among people with lived experience, in SCI care, research, and policy to create a promising future and sustain the strategy over the next decade	#/type of leaders recruited	More Skilled Workforce		
		Monitor and evaluate the strategy to ensure it has a real impact on Canadians	Annual updates on results			

Abbreviations: R&D, Research and Development; SCI, Spinal Cord Injury; SME, Small and Medium-sized Enterprises.
